# Choice Drivers for Quality-Labelled Food: A Cross-Cultural Comparison on PDO Cheese

**DOI:** 10.3390/foods10061176

**Published:** 2021-05-24

**Authors:** Davide Menozzi, Georges Giraud, Monia Saïdi, Ching-Hua Yeh

**Affiliations:** 1 Department of Food and Drug, University of Parma, Via Kennedy 6, 43125 Parma, Italy; 2 UMR CESAER, AgroSup Dijon, University Bourgogne Franche-Comté, 21000 Dijon, France; georges.giraud@agrosupdijon.fr (G.G.); monia.saidi@agrosupdijon.fr (M.S.); 3 Institute for Food and Resource Economics, University of Bonn, Nußallee 21, 53115 Bonn, Germany; chinghua.yeh@ilr.uni-bonn.de

**Keywords:** theory of planned behaviour, protected designation of origin (PDO) label, food quality schemes, intention, self-reported behaviour, discrete choice experiment

## Abstract

This study aims at examining the consumers’ preferences and drivers affecting the choice of quality-labelled food products, i.e., protected designation of origin (PDO) labelled cheese. We applied the theory of planned behaviour (TPB) to analyse the purchase of Parmigiano Reggiano PDO and Comté PDO hard cheeses in Italy and France, respectively. A cross-sectional sample of 808 consumers (400 French and 408 Italian) completed a questionnaire. Structural equation modelling (SEM) indicated perceived behavioural control (PBC) and attitude to be significant predictors of intention to purchase PDO-labelled cheese in France and Italy. Subjective and moral norms affected intention in France. Intention significantly influenced the hard cheese purchase behaviour. The results confirm that the TPB model predicted the self-reported measure of behaviour more than the observed one, measured with a discrete choice experiment, in both countries. The TPB interrelationships varied between countries, suggesting that food systems operators and public authorities should carefully target their intervention to stimulate the demand of PDO-labelled products.

## 1. Introduction

The EU food quality policy (EU Regulation n. 1151/2012) aims to protect the names of specific food and wines and to promote their unique intrinsic attributes and reputation strictly connected with their geographical origin, as well as traditional production methods. One of the specific objectives of the Protected Designations of Origin (PDO) and Protected Geographical Indications (PGI) labels is to provide clear information on products with particular characteristics linked to geographical origin, enabling consumers to make more informed purchasing choices [1,2]. Italy and France represent 38% of the total EU PDO and PGI recognitions. These products’ final market is estimated at EUR 14.4 bln in Italy [3] and EUR 4.1 bln in France, excluding beverages [4]. Among the PDO and PGI foods, cheeses represent a significant part, both in number of designations (54 in Italy and 55 in France), and in the value of the market. Indeed, the final market of PDO and PGI cheese is estimated at EUR 7.16 bln in Italy [3] and EUR 2.11 bln in France, excluding fresh cheese and cream [5].

The Parmigiano Reggiano (Parmesan) PDO cheese is a hard granular cheese produced in a limited number of Northern Italian provinces with strict farming and processing rules [6,7,8,9]. The production in 2018 was 144 thousand tons with a final market value of EUR 2.44 bln, representing alone approx. 34% of the PDO cheese market value [3]. The exports now account for approx. 41% of the production [8]. The Comté PDO cheese is a raw milk, cooked and pressed cheese, produced in the Jura Mountains over three French departments. The overall production is approx. 68 thousand tons and the market is mostly national, and more than 90% of sales are in France [10].

It is generally recognised the importance of the designations of origin in improving the communication, between producers and consumers, of the product attributes and production methods [1,11], as well as the transparency of the market, by reducing moral hazard risks [12,13]. Consumers’ awareness of geographical indications (GIs), in particular PDO food products, is higher in the countries with a stronger market tradition of such quality schemes, such as Italy, France, and Spain, compared to Northern European countries [1,14]. In particular, the interest in the origin of foods and in receiving information about product quality through a PDO/PGI label, and the belief that PDO/PGI signal better quality, are important determinants of consumers’ intention to purchase and willingness to pay (WTP) for the protected PDO/PGI product [1,11,15]. Besides the quality warranty, the social dimension of the PDO labels, including the social and territorial contexts inside which the food is produced, stored, sold and more broadly, conceived, may affect consumers’ attitude [15]. In a study conducted in Northern Italy, it was found that consumers who purchased a larger quantity of the PDO cheese in the aftermath of a natural disaster (i.e., the 2012 earthquake) were those who trusted the information provided by producers and retailers, perceived a stronger image of the PDO label, and a stronger sense of belonging to the region of origin [16]. Other studies have demonstrated the consumers’ perceived involvement with purchasing PDO-labelled products through short food supply chains. Consumers were encouraged to buy Parmigiano Reggiano PDO cheese in a local dairy shop in order to strengthen positive externalities at the local level, such as preserving local farming and, hence, stabilising local rural communities [8]. Furthermore, previous studies also reported that PDO products also allow us to draw lessons on adding value to, and protecting, traditional local products and know-how. The case of PDO Comté cheese in France shows how a PDO certification can generate public recognition of a product’s quality, therefore making it possible to increase the incomes of rural producers and to contribute to rural development [17].

This paper adds to the current knowledge by analysing the consumer determinants of intention to buy PDO-labelled food products, and the relative stated and observed buying behaviour. It also provides novel insights by assessing the consumers’ preferences and drivers affecting the choice of PDO-labelled food products in Italy and France, considering Parmigiano Reggiano PDO and Comté PDO hard cheeses as case studies. France and Italy were chosen as relevant countries since PDO cheeses represent a significant part of quality-label recognitions from a marketing perspective. Moreover, significant consumer behavioural differences have also been found across the two countries in other food-related contexts [18,19].

## 2. The Theoretical Framework

The present study applies the theory of planned behaviour (TPB) [20] to identify the main determinants of consumers’ choice of quality-labelled food, with specific reference to the PDO-labelled hard cheese purchase. The TPB is one of the most relevant theoretical frameworks used to analyse individuals’ behaviour. The TPB assumes that a given behaviour (e.g., choosing quality-labelled food) is guided by behavioural intentions, capturing the motivational dimension, and the perceived ability to perform the behaviour (perceived behavioural control, PBC). In turn, intentions are affected by individuals’ attitudes (i.e., favourable and unfavourable expected consequences of behaviour), subjective norms (i.e., the perceived social pressure in performing the behaviour), and by the PBC. More recently, Ajzen and Kruglanski suggested that the motivation to initiate a behaviour depends on the perceived likelihood or expectancy that performing the behaviour will bring about desired goals, as well as on the subjective values or magnitudes of these goals. They integrated the TPB with a theory about goals, because goals are central sources of motivation [21].

Prior applications of the TPB in predicting food consumption behaviour have shown that the TPB components are able to explain from 39 to 50% of the variance in intention and 27–36% of the variance in behaviour [22]. The potential of the TPB as a model for understanding sustainable behaviours was confirmed by a review conducted by Biasini and colleagues [23] which found a wide range of explained variance of intention (7–87%) and behaviour (3–81%). Another review found that the theory accounted for on average 21% of the variance predicted in health-oriented dietary behaviour [24]. In general, when considering discrete food choices, attitudes were found to have the strongest association with intention, followed by PBC and subjective norm, while the behaviour was mostly affected by intention and, to a lesser extent, by PBC [25]. From a cross-cultural perspective, another review highlighted that the impact of the TPB drivers, in particular the subjective norm, on intention may vary across countries [26]. Moreover, other studies have reported different consumers’ perceptions and behaviour in other food-related contexts [18,19]. Based on these considerations, this study suggests that:

***Hypothesis*** ***1.*** *A favourable attitude towards purchasing PDO-labelled products would significantly predict an increase in intention to buy PDO-labelled food products (i.e., hard cheese).*

***Hypothesis*** ***2.*** *Subjective norms would significantly predict intention to buy PDO-labelled food products.*

***Hypothesis*** ***3.*** *A higher PBC would significantly predict intention to buy PDO-labelled food products.*

***Hypothesis*** ***4.*** *Behavioural intentions to purchase PDO-labelled products would significantly predict the behaviour, i.e., PDO-labelled food products purchase.*

***Hypothesis*** ***5.*** *A higher PBC would significantly predict the behaviour, i.e., PDO-labelled food products purchase.*

Additional psycho-social variables have been often added to the TPB either as background factors influencing individuals’ beliefs or as additional contributors to the prediction of behavioural intention and behaviour [16,27,28]. Fishbein and Ajzen argue that, for certain classes of behaviour with a moral dimension, the nature of normative influence in the context of the TPB framework can be expanded and clarified by adding the concept of moral norms [29]. Moral norms are defined as the individual’s conviction of the moral correctness/incorrectness of behaving in a certain way [30], and are related with the “perceived social pressures [and] personal feelings of moral obligation or responsibility to perform, or refuse to perform, a certain behaviour” [20]. When moral norms have been included as additional predictors in the TPB, they have generally increased the proportion of explained variance in purchase intention of socially responsible products [31]. In accordance with these arguments, we have formulated the following additional hypothesis:

***Hypothesis*** ***6.*** *Moral norms are direct determinants of intention to buy PDO-labelled food products.*

According to the EU food quality policy, the PDO and PGI labels ensure reliable consumer information with regard to origin and quality of the products [32], enabling consumers to trust and distinguish higher quality food products from conventional ones. The role of the institutions, media, and peer-to-peer information in shaping consumers trust in the properties of the Parmigiano Reggiano PDO cheese was analysed, and found to be relevant in building consumers’ confidence in the PDO labelling schemes, affecting their intention to buy and their actual purchase of the PDO-labelled food [8,16]. Given these premises, we added the following hypothesis:

***Hypothesis*** ***7.*** *Trust in the EU PDO label would significantly predict intention to buy PDO-labelled food products.*

When considering behaviour, cross-sectional studies often consider a self-report measure of past or current behaviour, failing to meet the criterion of causality that would require a prospective measure of behaviour [29]. However, assuming that behaviour would be relatively stable over time, present intentions are likely to reflect past experience, and it is often assumed that they correlate better with a retrospective, than with a prospective measure of behaviour [29]. Indeed, other reviews have shown that intentions correlate better with self-reports than with more objective measures of behaviour [23,24,29]. In this paper, we applied two different behaviour measures: a self-reported measure of past behaviour, and a more objective measure of behaviour based on a discrete choice experiment (DCE) conducted with the two products, Parmigiano Reggiano PDO in Italy and Comté PDO in France. Therefore, we have formulated the following hypothesis:

***Hypothesis*** ***8.*** *The TPB model would better predict the self-reported past behaviour than the more objectively measured behaviour (i.e., the discrete choice).*

Fishbein and Ajzen argued that frequency of past behaviour accounts for appreciable variance in intentions even after controlling for attitudes, subjective norms, and perceived control, and has a large residual effect on prospective behaviour after controlling for intentions and PBC [29]. Given these arguments, we have added the following hypotheses:

***Hypothesis*** ***9.*** *Past behaviour would significantly predict intention to buy PDO-labelled food products.*

***Hypothesis*** ***10.*** *Past behaviour would significantly predict the current behaviour, i.e., PDO-labelled food products discrete choice.*

**Figure 1:**
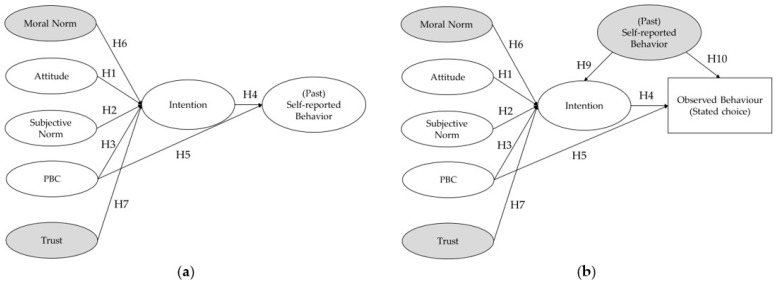
Hypothesised models: (**a**) model predicting a self-reported (past) behaviour; (**b**) model predicting an observed measure of behaviour (stated choice). In Model b2 the role of self-reported past behaviour was tested as predicting intentions and behaviour. In addition, ***Hypothesis 8*** postulates that the coefficient of determination (*R*^2^) of the behaviour in Model a is greater than the one in Model b. The variables added to the TPB original model are displayed in grey.

Therefore, this paper aims to confirm the TPB model predictors of purchasing quality-labelled food products, considering the case of PDO hard cheese in France and Italy. Materials and methods are described in next section. Two different models were tested (Figure 1), considering a self-reported measure of past behaviour (Model a), and a more objective measure of behaviour (Model b). In Model b the role of self-reported past behaviour was tested as predicting intentions and behaviour (Model b1 without past behaviour, and Model b2 with past behaviour). This approach adds knowledge to the current literature in several ways. It provides further evidence of the role of psychosocial determinants (attitude, subjective norms, and PBC) in explaining food behavioural choices in a cross-country perspective. Secondly, it adds further evidence of the different ability of the TPB in predicting self-reported and more objective measures of behaviour.

## 3. Materials and Methods

### 3.1. Data Collection and Sample

Data were collected during summer 2018 through a random and nationwide online survey administered to a population defined as adult shoppers above 18 years of age, living in France and Italy. Respondents had to be at least partly responsible for their household food shopping, and have bought cheese in the last three months. A third party research institute, LiGHTSPEED, collected the data online using its consumer panel database (the survey questionnaire is available upon request). The research was part of the Strength2Food project (H2020, Grant Agreement n. 678024), and received ethical approval from the coordinating institution (Newcastle University); data collection, handling, and storage procedures were also approved by the European Commission prior to commencement of the research.

The final sample in each country consisted of approximately 400 consumers (808 in total), biased in favour of respondents being wealthier for the French data in relation to the Italian data. The main sample characteristics are reported in Table 1. In general, participants were half female and mostly well-educated. In France they were equally living in the rural and urban areas, whereas in Italy they were mostly living in the urban area (medium and large cities). The mean age was 40 years for France and 43 years for Italy. Household size was slightly larger in Italy (3.1 vs. 2.6 members).

### 3.2. Measures

The questionnaire items were defined according to the TPB conceptual and methodological considerations [20,29] and the previous findings on similar topics (see Appendix A Table A1). We assessed the direct measure of attitude toward the behaviour with six semantic differentials, using a 7-point semantic scale, e.g., “Buying PDO-labelled hard cheese instead of hard cheese without such a label would make me feel unsatisfied/satisfied”, and “I think that buying PDO-labelled hard cheese instead of hard cheese without such a label is meaningless/meaningful”. It is generally recommended to include a measure of social norms that incorporates both injunctive and descriptive norms in empirical analysis [29]. Therefore, as a direct measure of subjective norms we used two items of injunctive norms on a 7-point Likert scale (e.g., “Most people who are important to me would like me to buy PDO-labelled hard cheese instead of hard cheese without such a label”), and one item of descriptive norms (“Most of my close friends and family generally buy PDO-labelled hard cheese instead of hard cheese without such a label”). We directly measured perceived behavioural control (PBC) with two items, on a 7-point scale, e.g., “Whether or not I buy PDO-labelled hard cheese instead of hard cheese without such a label on a regular basis is completely up to me”. Trust in the PDO label was assessed with three items measured on a 7-point Likert scale, such as “Products with the EU PDO label fulfil strict rules”, while we measured moral norm with three items (e.g., “Buying PDO-labelled hard cheese instead of hard cheese without such a label: would feel like I am making a personal contribution to something better”). We used three items to assess behavioural intention, e.g., “I intend to buy PDO-labelled hard cheese instead of hard cheese without such a label on a regular basis”. Positive and negative endpoints were counterbalanced throughout the questionnaire to avoid possible systematic response set.

The behaviour of interest was measured considering both past behaviour and prospective stated choices by respondents. Past behaviour was self-reported by consumers, responding to five different items using a 7-point scale, such as, “When you buy hard granular cheese, how often do you buy hard granular cheese with a PDO label”, “On average, how often do you buy hard granular cheese?”, and “On average, how often do you eat hard granular cheese?”. Moreover, a more objective measure of behaviour was assessed with a discrete choice experiment (DCE) [33]. By conducting an internal discussion among academic researchers and market experts, we defined three attributes, namely Quality Label (levels: no-label generic hard cheese; PDO Comté/Parmigiano Reggiano, and PDO organic-labelled Comté/PDO Product of the Mountain Parmigiano Reggiano), Brand (no brand/large-scale retailer’s brand; farm’s brand/national brand; and cheese refiner brand/local brand) and Price. The DCE applied in the present study has an unlabelled design, with three alternatives and an opt-out alternative. More details of the experimental design can be found in [33]. The discrete choice behaviour variable was the simulated utility estimated by taking the average of the utilities a consumer obtains from buying PDO cheese (with a PDO Comté/Parmigiano Reggiano, or a PDO organic-labelled Comté/PDO Product of the Mountain Parmigiano Reggiano) with all branded conditions at all four prices considered in the DCE. Thus, for each consumer a normalised individual utility value for buying PDO cheese was estimated and added to the SEM.

The questionnaire was first developed in English, and then translated into French and Italian. We used a back-translation method to avoid semantic discrepancies (e.g., translation errors, different interpretations, etc.) between countries.

### 3.3. Data Analysis

The data were initially analysed to confirm correlations between the predictors (i.e., attitude, subjective norm, PBC, moral norm, and trust) with both intention and the behaviour. Mean values and standard deviation were calculated for each construct from the items’ scores. Then, item reliability (factor loadings, λ, and Cronbach’s α) and composite reliability (CR) were tested, whilst average variance extracted (AVE) was used to assess convergent validity. AVE measures the level of variance captured by a construct with respect to items due to measurement error. We can calculate the AVE value by first squaring the factor loadings of each item, adding these factor scores for each variable, and then dividing it by the number of items each variable has. The discriminant validity was tested by comparing the squared root of the AVE of each construct with inter-construct correlation [34]. We applied a structural equation modelling (SEM) technique to test the suggested model and hypothesis. SEM is a statistical methodology that takes a confirmatory (i.e., hypothesis testing) approach to the analysis of a structural theory on a specific phenomenon. A confirmatory factor analysis (CFA) was performed to test the ability of the measurement variables to be represented by a set of latent variables, and further to assess the measurement model for the validity as well as reliability of items measuring the SEM construct.

The goodness of fit of the model was assessed considering the comparative fix index (CFI), Tucker–Lewis index (TLI), standardised root mean square residual (SRMR), and root mean square error of approximation (RMSEA). These are among the fit statistics that should be reported, as generally recommended [35]. The coefficient of determination (*R*^2^) measured the explained variance of the endogenous variables (intention and behaviour). The model was estimated using the Mplus software; we operated the SEM applying the maximum likelihood estimator with robust standard errors (MLR) routine.

## 4. Results

Table 2 shows the CFA statistics performed for the TPB variables and added constructs. The factor loadings (λ) are above 0.50, with only a few exceptions in the Italian case, the CR values are between 0.70 and 0.94, Cronbach’s α is in the 0.68 to 0.93 range, and the AVE values are in the range of 0.33 to 0.83. According to Fornell and Larcker [36], if AVE is less than 0.5, but composite reliability (CR) is higher than 0.6, than the convergent validity of the construct is still considered adequate. Overall, these values indicate that all factors in the measurement model have strong reliability and convergent and discriminant validity.

### 4.1. Descriptive Statistics

The results in Table 2 show a positive attitude toward buying PDO-labelled hard cheese in France and Italy (mean scores 5.33 and 5.30, respectively), a moderately positive social pressure, slightly lower in France than in Italy (4.17 and 4.58, respectively), and a generally positive perceived control (5.05 and 5.29, respectively) (Table 2). Overall, respondents reported moderately positive (4.80) and positive (5.21) intentions to buy PDO-labelled hard cheese, respectively, in France and Italy. Respondents exhibited positive trust in the EU PDO labels in both countries (mean score 5.10 in France, and 5.29 in Italy), and moderately positive moral obligation in buying PDO-labelled hard cheese (4.92 and 4.80, respectively). Considering past behaviour, on average, consumers were indicated to more often buy hard cheese with a PDO label and to more often eat hard cheese in Italy than in France (items PB1 and PB5, respectively, Table 2). The utility score of the Discrete Choice Behaviour shows a relatively higher utility obtained from buying PDO cheese in Italy than in France.

All correlations between the TPB variables and added constructs are significant at *p* < 0.001 (Table 3), with the only exception being the correlation between attitude and past behaviour in Italy, which is marginally significant (*p* = 0.050). The discriminant validity was assessed since the squared root of AVE of each construct was greater than the correlation between constructs [34]. Attitude, subjective norms, and, especially, PBC are statistically significantly correlated with intentions in France (respectively, *r* = 0.53, 0.58, and 0.72) and in Italy (respectively, *r* = 0.31, 0.54, and 0.76). Moral norm and trust also correlate with intention in France (respectively, *r* = 0.63 and 0.46) and Italy (respectively, *r* = 0.58 and 0.45). The self-reported measure of past behaviour is significantly correlated with intention and PBC, in France (respectively, *r* = 0.46 and 0.42) and Italy (respectively, *r* = 0.33 and 0.25), whereas the observed measure of choice behaviour (i.e., the discrete choice utility score) is significantly correlated with intention and PBC, in France (respectively, *r* = 0.46 and 0.43) and Italy (respectively, *r* = 0.20 and 0.15).

### 4.2. Predicting Intentions and the Behaviour

**Figure 2:**
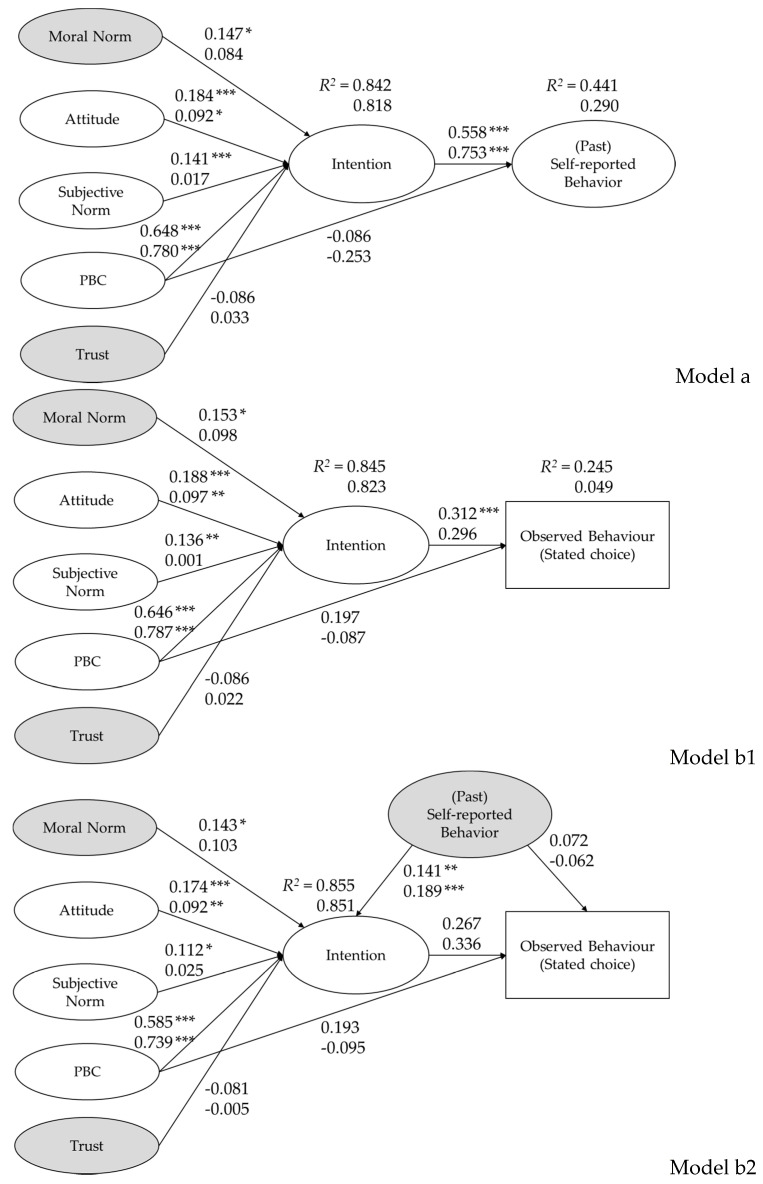
Model a, Model b1, and Model b2, predicting Behavioural Intention, Past (self-reported) Behaviour, and Observed Behaviour (Discrete Stated Choice) in France (*n* = 400) and Italy (*n* = 408). Standardised coefficients are displayed (France = upper value; Italy = lower value); Sig.: *** *p* < 0.001; ** *p* < 0.01; and * *p* < 0.05.

Table 4 and Figure 2 show the results of the three tested models. The hypothesised models fit the data well in both countries, as shown by the fit indices (Table 4) [35].

Model a is able to predict 84 and 82% of the intention to purchase PDO-labelled hard cheese, respectively, in France and Italy, and 44 and 29% of self-reported behaviour. Intention is the only significant predictor of the self-reported behaviour in France and Italy (respectively, β = 0.56, *p* < 0.001, and β = 0.75, *p* < 0.001), therefore confirming ***Hypothesis 4***. ***Hypothesis 5***, regarding the significant effect of PBC in affecting the behaviour, is not supported by the data. However, PBC is the main predictor of intention in both countries (respectively, β = 0.65, *p* < 0.001, and β = 0.78, *p* < 0.001), supporting ***Hypothesis 3***. The effect of subjective norm on intention is significant in France (β = 0.14, *p* < 0.01), supporting ***Hypothesis 2***, whereas this hypothesis is not confirmed in Italy. Attitude is a significant predictor of intention in France and Italy (respectively, β = 0.18, *p* < 0.001, and β = 0.09, *p* < 0.05), confirming ***Hypothesis 1***. Moral norm affects intention to purchase PDO-labelled hard cheese in France (β = 0.15, *p* < 0.05), supporting ***Hypothesis 6***. This hypothesis is not confirmed, however, in Italy. In contrast with ***Hypothesis 8***, the effect of trust on intention is not statistically significant in both countries. From a cross-cultural perspective, the ambivalent effect of subjective norms and moral norms on affecting intention in France compared to Italy, suggests some variability across countries. 

Based on the *R*^2^, the Model b1 explains 85 and 82% of the variance in intention, and 25 and 5% of the variance in the observed (discrete choice) behaviour in France and Italy, respectively. These results support ***Hypothesis 8***, showing that the TPB model better predicts the self-reported past behaviour than the more objective measure of behaviour (i.e., the discrete choice observation). Still, intention is the only significant predictor of the behaviour in France (β = 0.31, *p* < 0.001), confirming ***Hypothesis 4***, whereas it does not significantly affect the behaviour in Italy. ***Hypothesis H5*** (PBC → behaviour) is again not supported by the data. The significant predictors of the intention are the same as in Model a in both countries, with similar coefficients. Therefore, ***Hypothesis 1*** (attitude → intention) and ***Hypothesis 3*** (PBC → intention) are supported in both countries, ***Hypothesis 2*** (subjective norm → intention) and ***Hypothesis 6*** (moral norms → intention) are only confirmed in France, and ***Hypothesis 7*** (trust → intention) is not supported neither in France nor in Italy. The interrelationships between subjective and moral norms variables, and the different effects of the intention on the behaviour across countries, again suggest some cross-cultural differences.

When past behaviour is added as predicting intention and observed behaviour (Model b2), it shows a positive and significant effect on intention to purchase PDO-labelled hard cheese in France and Italy (respectively, β = 0.14, *p* < 0.01, and β = 0.19, *p* < 0.001), supporting ***Hypothesis 9***. By adding past behaviour to the model, an additional 1 and 3% of the variance in intention can be explained, respectively, in France and Italy, resulting in a final *R*^2^ of 0.86 and 0.85. However, ***Hypothesis 10*** is not confirmed, since past behaviour does not significantly affect observed behaviour; in this case, the explained variance does not improve neither in France, nor in Italy.

## 5. Discussion

The aim of the present study was to examine the consumers’ preferences and drivers affecting the choice of quality-labelled food products, considering the case of Parmigiano Reggiano PDO and Comté PDO hard cheeses in Italy and France, respectively. Our findings suggest that perceived behavioural control and, to a lesser extent, attitude play a significant role in affecting the intention of performing the behaviour in France and Italy. Instead, the subjective norm, although being significantly correlated with intentions in both countries, is a significant factor in forming the behavioural intention only in France. Therefore, in this study we found that the opinions and behaviour of salient others, such as family and friends, only marginally affected the intention in France, whereas their effect in Italy was not significant. These results confirm those of another study, investigating the purchase of Parmigiano Reggiano PDO in the aftermath of a natural disaster, where only PBC and, marginally, attitude were significant predictors of intention, whereas the effect of subjective norm was not statistically significant [16]. Another review also showed the weakest effect of subjective norms in predicting intention, as compared to attitude and PBC, when considering discrete food choice behaviours [25]. The ambivalent effect of subjective norms in a cross-cultural perspective was also confirmed by a review showing that the impact of subjective norm on intention may vary most across countries, whereas the relationship between intention and both attitude and PBC operates more similarly across country samples [26].

Moral norms were also found to be significant predictors of intention in France; they were generally considered meaningful for those behaviours that have a moral dimension, expanding and clarifying the nature of the normative component in the context of the TPB [29]. Others authors suggested to maintain them separate from the subjective norms, since they embrace the personal norms, rather than the social pressure to perform the behaviour; in this way they accounted for an additional effect after controlling for the TPB variables [31]. Moral norms do not significantly predict intentions in Italy, although the correlation is significant; this might mean that the causal effect of PBC in affecting intention in that country is so strong as to overcome the other effects. Personal moral norms were also found to be significantly correlated with intention to buy Parmigiano Reggiano PDO cheese, but not with the behaviour, in another study [16].

In the present study, although being correlated with intention and behaviour, trust was not reported as a significant predictor. A higher trust in the EU PDO labelling system is significantly positively correlated with intention to purchase PDO cheese and behaviour in both countries; however, it fails to affect these variables when the causal effect is considered. This contradicts the results of other studies. In one case study conducted in France and Italy, when trust in the traceability system was added to the model it became the most significant predictor of intention to purchase traceable food, in Italy for honey and chicken, and in France only for honey [18]. In the UK, trust in food safety information as provided by different sources significantly reduced the likelihood to purchase chicken meat, indicating that, when food scare occurs, trust in information provided by media is able to amplify the negative effects of likelihood to purchase [37].

Intention is the most important predictor of both self-reported and observed behaviour in France, whereas it only affected the self-reported behaviour in Italy. The more significant effect of intention over PBC in predicting discrete food choice behaviours has been also tested by different meta-analyses [22,25]. The results, overall, indicate that the motivational component is the only one affecting the consumers’ behaviour, both self-reported and observed in the discrete choice experiment. The perceived control, indicating the personal perceived ability to purchase PDO-labelled cheese, has only a significant effect on intentions. In other words, feeling able to purchase the quality-labelled food has an influence on an individual’s intention to buy it and, by means of this motivation, makes the behaviour more likely to occur. The wide availability and high penetration of these PDO-labelled products in the respective markets may have reduced the perceived barriers’ ability to act as obstacles to the respondents’ purchases, once the intention to perform the behaviour was expressed.

Cross-cultural differences were found in the present study. The TPB variables together with moral norms and trust accounted for a quite uniform explained variance in intention in France and Italy, i.e., in the range of 82 to 86%. However, the behaviour, both self-reported and more objectively observed with the DCE, was better predicted by intention and PBC in France than in Italy (self-reported: 44 vs. 29%; DCE: 25 vs. 5%). These differences may be explained by a diverse cultural approach to the purchase of PDO-labelled cheese as expressed by the TPB in the two countries. In France, the market share of Comté is very high among both PDO-PGI cheeses and all cheese purchases, respectively, 28.4 and 12.6%. This sustains a good recall of Comté cheese in consumers’ minds. Consequently, the intention to buy and the self-reported buying behaviour with respect to Comté is higher than the DCE measurement, which was carried out as an online experiment and not as acts of daily life. Socio-demographic variables, as well as non-cognitive factors, such as degree of acculturations, habits, and emotions, could be assessed and applied as moderators to improve the understanding of behaviour, especially in Italy.

Nevertheless, this study has confirmed that the self-reported measure of behaviour is better predicted than the observed one, in both countries. Lower predictive power is common in studies addressing more objective, rather that self-reported, behaviour [23,24,29]. This may be explained by the stronger measurement correspondence when self-reported measures are used; as in the present study, usually the self-reported measure of behaviour is more in line with the measure of intention, whereas the observed measure is not [22]. Despite this discrepancy, others have suggested to combine subjective and objective behavioural assessments to identify potential gaps between the self-perception of behaviour and its actual performance [23]. When modelling past behaviour as a predictor of intentions and observed behaviour, we have shown that it accounts for additional variance in intentions, even after controlling for attitudes, subjective norms, moral norms, trust, and PBC, incrementing its predictive power of 1 and 3% in France and Italy, respectively. However, it does not account for any residual effect on observed behaviour. In other words, we did not find any significant effect of past behaviour in directly affecting consumers’ stated choices, its effect being mediated by intentions. The results confirm the arguments suggesting a significant direct effect of past behaviour on intentions, even after taking into account the other variables [29].

We need to address some limitations of this study. First, we adopted a cross-sectional study design; even though this is quite common in TPB studies, being more feasible and less resource-intensive compared to longitudinal design [23], we were not able to provide a prospective prediction of the behaviour, and we were not in a position to analyse the causal relationship between dependent and independent variables [24]. Therefore, we did not have a perfect compatibility of behaviour with their antecedents; this might have deflated the predictive power of the model, in particular when the observed measure was applied. Secondly, this observed measure is based on a hypothetical experiment, which did not imply an actual purchase decision by respondents. However, the introduction of a cheap talk at the beginning of the experiment should have minimised the hypothetical bias [38]. Then, we applied also a self-reported measure of past behaviour as an endogenous variable. However, the market penetration of Parmigiano Reggiano PDO is stably high in Italy [13], as well as the Comté PDO in France [5]. Therefore, we feel fairly confident that our self-reported measure of past cheese consumption would not have changed much if it was assessed longitudinally.

## 6. Conclusions

We found that PBC and attitude were the main drivers of the intention to purchase PDO-labelled cheese in France and Italy, and that intention, in turn, significantly affects self-reported and observed behaviour. We also confirmed that the self-reported measure of behaviour is better predicted by the TPB model in both countries than the observed one, measured with the discrete choice experiment. We evidenced the differences that underline the TPB interrelationships between countries, showing the significant effect of subjective and moral norms in influencing the intention to purchase the PDO-labelled cheese in France. These results can be used by food systems operators (e.g., producers, retailers, Consortia, etc.), as well as by public authorities, as leverage points for influencing quality food consumption and increasing the demand for PDO-labelled products.

## Figures and Tables

**Table 1:** Sample structure (all data in percentage, apart from age, household size, and number of children).

Socio-Demographic Classes and Levels		France	Italy	All
		*n* = 400	*n* = 408	*n* = 808
Food purchase responsibility	Mainly responsible	72.0	63.7	67.8
	Partly responsible	28.0	36.3	32.2
Gender	Female	50.0	49.8	49.9
	Male	50.0	50.2	50.1
Age	Average years	40.0	42.9	41.5
Living area	Rural area	49.5	12.7	30.9
	Urban medium town	25.0	41.9	33.5
	City	25.5	45.3	35.5
Education	Lower secondary/primary or below	4.5	7.1	5.8
	Upper secondary education	31.8	38.5	35.1
	University or college entrance qualification	27.5	16.4	21.9
	Bachelor’s degree or equivalent level	20.5	16.4	18.4
	Master, Postgraduate, or doctoral degree	15.8	21.6	18.7
Household monthly net income	(FR) < EUR 1130/(IT) < EUR 900	11.5	7.1	9.3
	(FR) EUR 1131–1450/(IT) EUR 901–1500	6.5	18.4	12.5
	(FR) EUR 1451–2090/(IT) EUR 1501–2500	20.8	30.9	25.9
	(FR) EUR 2091–2890/(IT) EUR 2501–3500	18.5	21.6	20.0
	(FR) EUR 2891–4100/(IT) EUR 3501–4500	24.5	5.9	15.1
	(FR) ≥ EUR 4101/(IT) ≥ EUR 4501	12.5	1.7	7.1
	Prefer not to answer	5.8	14.5	10.1
Household size	Number of persons	2.6	3.1	2.9
Children	Number of children	0.6	0.5	0.6

**Table 2:** Descriptive analysis: mean, standard deviation (SD), standardised factor loadings (λ), Cronbach’s alpha (α), composite reliability (CR), and average variance extracted (AVE), in France (*n* = 400) and Italy (*n* = 408).

Constructs and Items	France						Italy					
	Mean	SD	λ	α	CR	AVE	Mean	SD	λ	α	CR	AVE
Attitude	5.33	1.11	-	0.87	0.83	0.47	5.30	1.17	-	0.86	0.82	0.50
A1	5.54	1.26	0.50 ***				5.38	1.50	0.35 ***			
A2	5.19	1.54	0.52 ***				5.06	1.68	0.44 ***			
A3	5.33	1.50	0.52 ***				5.16	1.66	0.47 ***			
A4	5.31	1.22	0.83 ***				5.42	1.39	0.76 ***			
A5	5.36	1.51	0.75 ***				5.28	1.52	0.88 ***			
A6	5.24	1.48	0.89 ***				5.49	1.45	0.93 ***			
Subjective Norm	4.17	1.51	-	0.89	0.90	0.75	4.58	1.36	-	0.87	0.87	0.70
SN1	4.20	1.69	0.89 ***				4.64	1.58	0.89 ***			
SN2	4.05	1.75	0.93 ***				4.49	1.58	0.88 ***			
SN3	4.27	1.55	0.76 ***				4.62	1.44	0.73 ***			
PBC	5.05	1.03	-	0.76	0.77	0.53	5.29	1.14	-	0.84	0.84	0.63
PBC1	5.27	1.22	0.50 ***				5.41	1.32	0.69 ***			
PBC2	4.98	1.26	0.87 ***				5.26	1.31	0.87 ***			
PBC3	4.90	1.28	0.78 ***				5.18	1.31	0.81 ***			
Intention	4.80	1.16	-	0.87	0.89	0.73	5.21	1.16	-	0.88	0.88	0.71
BI1	4.88	1.29	0.86 ***				5.29	1.33	0.84 ***			
BI2	4.84	1.39	0.82 ***				5.19	1.38	0.88 ***			
BI3	4.70	1.16	0.88 ***				5.16	1.18	0.80 ***			
Trust	5.10	1.13	-	0.91	0.91	0.78	5.29	1.25	-	0.93	0.93	0.83
T1	5.21	1.18	0.90 ***				5.34	1.28	0.90 ***			
T2	5.09	1.24	0.87 ***				5.34	1.32	0.92 ***			
T3	5.00	1.27	0.87 ***				5.19	1.40	0.91 ***			
Moral Norm	4.92	1.16	-	0.88	0.89	0.73	4.80	1.33	-	0.92	0.92	0.80
MN1	5.06	1.23	0.90 ***				4.96	1.40	0.88 ***			
MN2	5.15	1.25	0.91 ***				4.84	1.44	0.89 ***			
MN3	4.56	1.39	0.73 ***				4.61	1.45	0.90 ***			
Past Behaviour	3.83	1.48	-	0.78	0.78	0.42	4.06	1.37	-	0.68	0.69	0.31
PB1	4.42	1.33	0.83 ***				5.23	1.16	0.75 ***			
PB2	4.57	1.21	0.62 ***				4.73	1.36	0.48 ***			
PB3	3.40	1.65	0.63 ***				3.24	1.66	0.36 ***			
PB4 ^1^	3.18	1.41	0.61 ***				3.21	1.34	0.59 ***			
PB5 ^1^	3.34	1.42	0.51 ***				3.91	1.32	0.55 ***			
Discrete Choice Behaviour ^2^	−0.17	56.80					16.70	49.50				

^1^ 6-point scale. ^2^ France: min = −110.1; max = 123.5. Italy: min = −117.9; max = 119.8. Sig.: *** *p* < 0.001. The measures are reported in Appendix A Table A1.

**Table 3:** Square root of AVE (diagonal elements) and inter-construct correlations, in France (*n* = 400) and Italy (*n* = 408).

Constructs	Country	ATT	SN	PBC	MN	Trust	BI	PB	DCB
ATT	FR	0.686	0.298	0.447	0.492	0.354	0.534	0.250	0.274
	IT	0.678	0.227	0.306	0.337	0.303	0.313	0.097	0.118
SN	FR		0.866	0.486	0.409	0.315	0.584	0.376	0.338
	IT		0.837	0.571	0.527	0.315	0.541	0.268	0.129
PBC	FR			0.728	0.550	0.483	0.721	0.419	0.433
	IT			0.794	0.547	0.463	0.760	0.251	0.147
MN	FR				0.854	0.582	0.632	0.357	0.310
	IT				0.843	0.378	0.576	0.243	0.171
Trust	FR					0.883	0.458	0.257	0.214
	IT					0.911	0.450	0.247	0.166
BI	FR						0.854	0.458	0.455
	IT						0.894	0.330	0.200
PB	FR							0.648	0.279
	IT							0.557	0.076
DCB	FR								1.000
	IT								1.000

ATT = Attitude; SN = Subjective Norm; PBC = Perceived Behavioural Control; MN = Moral Norm; BI = Behavioural Intention; PB = Past Behaviour; DCB = Discrete Choice Behaviour. Sig.: all correlations are significant at *p* < 0.001 (Attitude ↔ Past Behaviour in Italy, *p* = 0.050).

**Table 4:** Model a, Model b1, and Model b2, predicting Behavioural Intention (BI), Past Behaviour (PB), and Discrete Choice Behaviour (DCB) in France (*n* = 400) and Italy (*n* = 408) (*R*^2^ = coefficient of determination; beta = unstandardised coefficients; S.E. = standard error; *p* = *p*-values).

Model	Constructs	France				Italy			
		*R* ^2^	Beta	S.E.	*p*	*R^2^*	Beta	S.E.	*p*
Model a	BI predictors:	0.842				0.818			
	ATT		0.319	0.095	0.000		0.193	0.097	0.015
	SN		0.104	0.039	0.000		0.014	0.053	0.799
	PBC		1.182	0.216	0.000		0.957	0.130	0.000
	MN		0.147	0.068	0.029		0.076	0.072	0.292
	Trust		−0.090	0.053	0.090		0.033	0.050	0.510
	PB predictors:	0.441				0.290			
	BI		0.553	0.174	0.000		0.583	0.225	0.003
	PBC		0.211	0.300	0.482		−0.240	0.252	0.329
Model b1	BI predictors:	0.845				0.823			
	ATT		0.325	0.097	0.001		0.204	0.097	0.009
	SN		0.101	0.039	0.010		0.001	0.053	0.982
	PBC		1.174	0.216	0.000		0.965	0.137	0.000
	MN		0.152	0.067	0.022		0.089	0.071	0.214
	Trust		−0.091	0.053	0.090		0.021	0.052	0.676
	DCB predictors:	0.245				0.049			
	BI		16.042	7.564	0.000		13.104	8.082	0.099
	PBC		18.369	14.203	0.189		−4.705	9.751	0.630
Model b2	BI predictors:	0.855				0.851			
	ATT		0.301	0.094	0.001		0.193	0.089	0.009
	SN		0.083	0.039	0.034		0.020	0.050	0.680
	PBC		1.059	0.204	0.000		0.907	0.136	0.000
	MN		0.143	0.066	0.027		0.094	0.068	0.172
	Trust		−0.085	0.051	0.100		−0.005	0.051	0.923
	PB		0.143	0.052	0.007		0.243	0.059	0.000
	DCB predictors:	0.248				0.051			
	BI		13.725	7.808	0.079		14.856	9.337	0.107
	PBC		17.971	13.930	0.191		−5.171	10.342	0.618
	PB		3.720	4.051	0.357		−3.540	4.822	0.474

ATT = Attitude; SN = Subjective Norm; PBC = Perceived Behavioural Control; MN = Moral Norm; BI = Behavioural Intention; PB = Past Behaviour; DCB = Discrete Choice Behaviour. Model a fit indices: France: *χ*^2^ (DF) = 750.80(301) ***, TLI = 0.900; CFI = 0.914; RMSEA (90% C.I.) = 0.061(0.056–0.067); SRMR = 0.089. Italy: *χ*^2^ (DF) = 482.04(301) ***, TLI = 0.957; CFI = 0.963; RMSEA (90% C.I.) = 0.038(0.032–0.045); SRMR = 0.053. Model b1 fit indices: France: *χ*^2^ (DF) = 326.34(186) ***, TLI = 0.958; CFI = 0.966; RMSEA (90% C.I.) = 0.043(0.036–0.051); SRMR = 0.045. Italy: *χ*^2^ (DF) = 247.83(186) ***, TLI = 0.981; CFI = 0.985; RMSEA (90% C.I.) = 0.029(0.018–0.038); SRMR = 0.033. Model b2 fit indices: France: *χ*^2^ (DF) = 657.39(294) ***, TLI = 0.917; CFI = 0.930; RMSEA (90% C.I.) = 0.056(0.050–0.061); SRMR = 0.051. Italy: *χ*^2^ (DF) = 460.15(294) ***, TLI = 0.960; CFI = 0.966; RMSEA (90% C.I.) = 0.037(0.031–0.044); SRMR = 0.042. Sig.: *** *p* < 0.001.

## Data Availability

The data presented in this study are available on request from the corresponding author.

## Appendix A

**Table A1:** Questionnaire items, scales (in square brackets), and sources.

Construct	Code	Item	Sources
Attitude (ATT)		Buying PDO-labelled hard cheese instead of hard cheese without such a label would make me feel:	[29,39]
	A1	1 = unsatisfied; 7 = satisfied	
	A2	1 = unhappy; 7 = happy	
	A3	1 = bad; 7 = good	
		I think that buying PDO-labelled hard cheese instead of hard cheese without such a label is:	
	A4	1 = meaningless; 7 = meaningful	
	A5	1 = harmful; 7 = beneficial	
	A6	1 = unimportant; 7 = important	
Subjective Norms (SN)	SN1	Most people who are important to me would like me to buy PDO-labelled hard cheese instead of hard cheese without such a label (1 = strongly disagree, 7 = strongly agree)	[29]
	SN2	My close friends and family expect me to buy PDO-labelled hard cheese instead of hard cheese without such a label (1 = strongly disagree, 7 = strongly agree)	
	SN3	Most of my close friends and family generally buy PDO-labelled hard cheese instead of hard cheese without such a label (1 = strongly disagree, 7 = strongly agree)	
Perceived Behavioural Control (PBC)	PBC1	Whether or not I buy PDO-labelled hard cheese instead of hard cheese without such a label on a regular basis is completely up to me (1 = strongly disagree, 7 = strongly agree)	[29]
	PBC2	I am confident that I can buy PDO-labelled hard cheese instead of hard cheese without such a label on a regular basis (1 = strongly disagree, 7 = strongly agree)	
	PBC3	For me buying PDO-labelled hard cheese instead of hard cheese without such a label on a regular basis is easy (1 = strongly disagree, 7 = strongly agree)	
Behavioural Intention (BI)	BI1	I intend to buy PDO-labelled hard cheese instead of hard cheese without such a label on a regular basis. (1 = extremely unlikely, 7 = extremely likely)	[29]
	BI2	I will make an effort to buy PDO-labelled hard cheese instead of hard cheese without such a label on a regular basis. (1 = strongly disagree, 7 = strongly agree)	
	BI3	In the future when you buy hard cheese how often will you buy PDO-labelled hard cheese? (1 = never, 7 = every time)	
Trust in Label (T)	T1	Products with the EU PDO label fulfil strict rules (1 = strongly disagree, 7 = strongly agree)	[40]
	T2	The EU PDO label guarantees that the products are of a higher quality (1 = strongly disagree, 7 = strongly agree)	
	T3	I have great trust in the control system behind the EU PDO label (1 = strongly disagree, 7 = strongly agree)	
Moral Norm (MN)		Buying PDO-labelled hard cheese instead of hard cheese without such a label…	[30,41]
	MN1	… would feel like I am making a personal contribution to something better (1 = strongly disagree, 7 = strongly agree)	
	MN2	… would feel like the morally right thing to do (1 = strongly disagree, 7 = strongly agree)	
	MN3	… makes me feel like a better person (1 = strongly disagree, 7 = strongly agree)	
Past Behaviour (PB)		When you buy hard granular cheese, how often do you…	[29]
	PB1	… buy hard granular cheese with a PDO label (1 = never, 7 = almost every time)	
	PB2	… buy hard granular cheese carries a brand (1 = never, 7 = almost every time)	
	PB3	… buy hard granular cheese at a farmers’ market or butcher (1 = never, 7 = almost every time)	
	PB4	On average, how often do you buy hard granular cheese? (1 = less than once a month, 6 = more than once a week)	
	PB5	On average, how often do you eat hard granular cheese? (1 = less than once a month, 6 = every day)	
